# Health Providers’ Perceptions of Clinical Trials: Lessons from Ghana, Kenya and Burkina Faso

**DOI:** 10.1371/journal.pone.0124554

**Published:** 2015-05-01

**Authors:** Vibian Angwenyi, Kwaku-Poku Asante, Abdoulaye Traoré, Lawrence Gyabaa Febir, Charlotte Tawiah, Anthony Kwarteng, Alphonse Ouédraogo, Sodiomon Bienvenue Sirima, Seth Owusu-Agyei, Egeruan Babatunde Imoukhuede, Jayne Webster, Daniel Chandramohan, Sassy Molyneux, Caroline Jones

**Affiliations:** 1 Department of Public Health Research, KEMRI/Wellcome Trust Research Programme (KWTRP), P.O. Box, 230–80108, Kilifi, Kenya; 2 Kintampo Health Research Centre (KHRC), P.O. Box 200, Kintampo, Ghana; 3 Centre National de Recherche et de Formation sur le Paludisme (CNRFP), 01 BP 2208, Ouagadougou 01, Burkina Faso; 4 European Vaccine Initiative (EVI),Universitäts Klinikum Heidelberg, Im Neuenheimer Feld 326, 69120, Heidelberg, Germany; 5 Disease Control Department, London School of Hygiene and Tropical Medicine (LSHTM), Keppel Street, WC1E 7HT, London, United Kingdom; 6 The Ethox Centre, Department of Public Health, University of Oxford, Old Road Campus, Headington, Oxford, OX3 7LF, United Kingdom; 7 The Centre for Clinical Vaccinology and Tropical Medicine, Nuffield Department of Medicine, University of Oxford, Old Road Campus, Headington, Oxford, OX3 7LF, United Kingdom; Pennsylvania State University College of Medicine, UNITED STATES

## Abstract

**Background:**

Clinical trials conducted in Africa often require substantial investments to support trial centres and public health facilities. Trial resources could potentially generate benefits for routine health service delivery but may have unintended consequences. Strengthening ethical practice requires understanding the potential effects of trial inputs on the perceptions and practices of routine health care providers. This study explores the influence of malaria vaccine trials on health service delivery in Ghana, Kenya and Burkina Faso.

**Methods:**

We conducted: audits of trial inputs in 10 trial facilities and among 144 health workers; individual interviews with frontline providers (n=99) and health managers (n=14); and group discussions with fieldworkers (n=9 discussions). Descriptive summaries were generated from audit data. Qualitative data were analysed using a framework approach.

**Results:**

Facilities involved in trials benefited from infrastructure and equipment upgrades, support with essential drugs, access to trial vehicles, and placement of additional qualified trial staff. Qualified trial staff in facilities were often seen as role models by their colleagues; assisting with supportive supervision and reducing facility workload. Some facility staff in place before the trial also received formal training and salary top-ups from the trials. However, differential access to support caused dissatisfaction, and some interviewees expressed concerns about what would happen at the end of the trial once financial and supervisory support was removed.

**Conclusion:**

Clinical trials function as short-term complex health service delivery interventions in the facilities in which they are based. They have the potential to both benefit facilities, staff and communities through providing the supportive environment required for improvements in routine care, but they can also generate dissatisfaction, relationship challenges and demoralisation among staff. Minimising trial related harm and maximising benefits requires careful planning and engagement of key actors at the outset of trials, throughout the trial and on its’ completion.

## Background

Over recent decades increased funding has been made available for malaria clinical trials (CTs), especially late stage vaccine trials, in sub-Saharan Africa [1, 2]. The public health systems in many of the countries in which these CTs have been implemented are weak with the quality of health services affected by several supply and demand side constraints including the high cost of care, poor physical accessibility of health facilities, drug stock-outs, lack of essential equipment, and shortages of health professionals [3–5].

A requirement for the ethical conduct of CTs is that participants in the trial receive standards of care which meet local and international guidelines such as Good Clinical and Laboratory Practices [6–8]. To conduct CTs in low resource settings, substantial investments are often required to upgrade service provision and health facilities to meet these standards. Investments in service provision and facility infrastructure can include the purchase of highly specialized medical equipment, the development of fully equipped laboratories, investment in transport and vaccine ‘cold chain’ systems, and the construction of additional buildings [7, 9–11]. To address health workforce challenges, CTs often hire additional personnel and provide training and incentives to those already in post [12, 13].

With such inputs, CTs have the potential to generate benefits for local communities and there is a growing body of literature documenting local community perceptions and experiences of CTs [2, 9, 10, 14–18]. These studies suggest CT benefits to communities include improved standards of care (ancillary care), better health outcomes due to continuous disease surveillance, and improved access to health facilities. At the research programme level, CT investments contribute to capacity development (both human resource and infrastructure) which in the long-run makes such centres become better contenders for future CTs [2, 11, 19]. However, for large multi-country studies, implementation challenges include translation of informed consent documents, community perceptions of risks and benefits, comprehension of technical procedures and community engagement strategies that are culturally acceptable [2, 9, 10, 14–18].

A few recent studies suggest that during their conduct, clinical trials can contribute to the quality of routine healthcare provided to trial participants [12, 20], and investments towards upgrading health facilities to CT standards create the necessary platform to attract and conduct other research in future [2, 11, 19]. On the other hand, anecdotal information suggests that clinical trials may negatively impact routine healthcare delivery by diverting resources. Little is known about if and how the inputs of CTs affect the provision of routine health care for non-participating children and adults attending CT facilities, or how the providers of routine care in such settings perceive and are affected by these inputs. Furthermore, little attention has been paid to potential post-trial effects on the provision of routine health care. Understanding the possible post-trial positive and negative consequences for routine care could contribute to changes in the way clinical trials are planned and implemented, ultimately strengthening ethical practice.

To increase our understanding of the impact of clinical trials on the delivery of health care to children in low resource settings, a consortium of African and European experts in CTs, social science, community engagement and public health was established in January 2011. The consortium was led by the Kintampo Health Research Centre (KHRC) in Ghana and involved partners from the KEMRI-Wellcome Trust Research Programme (KWTRP) in Kenya, the Centre National de Recherche et de Formation sur le Paludisme (CNRFP), Ouagadougou in Burkina Faso, and the London School of Hygiene and Tropical Medicine (LSHTM) in the UK. The consortium developed a multi-site, multi-component study whose overall aim was to gain insight into the impact of clinical trials on health services in three countries in sub-Saharan Africa (Ghana, Kenya and Burkina Faso), especially with regards to the quality of services delivered to children. We recognise that in many clinical trial settings, including those involved in the consortium, the expertise and resources that are required to conduct trials ethically mean that certain sites often run a number of trials over time and sometimes concurrently. Thus at any given point of time, health care in facilities may be influenced by historical and on-going trials. In this paper, we focus on the specific inputs and perceived impacts related to two major malaria vaccine trials in three sites. Although we recognise that these factors will be influenced by the trial context or ‘platform’ of research activities and relationships, and mention this where relevant, this platform is the focus of additional analysis currently in progress.

### Study setting and health system context

The public health systems in Ghana, Kenya and Burkina Faso are often constrained by inadequate basic health infrastructure, poor road networks, and other service delivery barriers such as drug shortages, long distances to facilities, high cost of treatment, and staff shortages [21, 22]. Further health workforce challenges, particularly for rural health facilities, include high staff workload, inappropriate skills-mix and high staff-patient ratio [23, 24]. In all three countries, total government expenditure on health as a percentage of gross domestic product is well below the 2001 Abuja declaration target of 15% [25], and the health systems depend largely on external donor funding and out-of-pocket payments through health insurance or at point of care payment [26, 27]. At the time of this study, national health sector reforms being implemented in the three countries included a health insurance scheme (NHIS) in Ghana, a direct facility funding initiative (HSSF) in Kenya, and a primary health care subsidy initiative to improve skilled delivery in Burkina Faso [28–30].

The KHRC in Ghana, the KWTRP in Kenya and the CNRFP in Burkina Faso are all well-established research centres conducting multi-disciplinary research on malaria and other infectious diseases in sub-Saharan Africa. Each of these research centres has established clinical trial facilities and a health demographic surveillance system (HDSS) from which potential-research participants are drawn. Each centre has over 5 years of experience in conducting malaria clinical trials [31–39]. These centres are all located in rural or peri-urban settings, characterised by low literacy and high poverty levels, with subsistence farming as the main economic activity (see Table 1).

**Table 1:** Research centers description.

Characteristics of trial centres	KHRC (Ghana)	KWTRP (Kenya)	CNRFP (Burkina Faso)
Year of establishment	1994	1989	1983
Location	Brong Ahafo Region, middle belt of Ghana	Kilifi County, Kenyan Coast	Ouagadougou
Institutional size (research capacity) year 2012/13	Over 600 staff (120 researchers)	Over 800 staff (77 researchers)	Over 150 staff (57 researchers)
Year clinical trial facility established	2005	2007	2009
Examples of malaria clinical trials conducted	RTS,S phase II and phase III malaria vaccine trials, Artemether-Lumefantrine malaria drug trial	RTS,S phase II and phase III malaria vaccine trials, TrapVac trial, ME-Trap vaccine trial, Artemether-Lumefantrine malaria drug trial	Phase IIb GMZ 2 malaria vaccine trial, Phase IIb ME-TRAP candidate malaria vaccine trial, Phase Ib MSP 3 candidate vaccine, Artemether-Lumefantrine malaria drug trial
Malaria transmission	High endemic region	Low endemic region	Hyper-endemic with seasonal malaria transmission
Poverty levels	25% of its population in the lowest wealth quintile	Over 60% of the population living below poverty lines	Estimated to be at 40%
Literacy levels	Over 52% with no formal education	About 45% of adults unable to read and write	Estimated to be at 21.8%
Main economic activities	Subsistence farming	Subsistence farming, small scale fishing and mining	Subsistence farming, craft industries, fishing and local manufacturing
Predominant group(s)	Akans (bonos) and Mos, Wangaras, Gonja and Mamprusi (Northern region)	Mijikenda	Goin, Karaboro and the Turka

In all three countries the research centres have a close collaboration with the Ministry of Health and provide support to health facilities where clinical research is undertaken. For instance, in Ghana the children’s ward in Kintampo North Municipal Hospital has over the years since the establishment of the KHRC received hospital equipment and upgrades from previous malaria clinical trials [31, 40]. In Kenya, through the MoH collaboration, KWTRP supports both children and adult wards in Kilifi County Hospital with the provision of medicines, supplies and diagnostic tests [20, 41]. In Burkina Faso, part of the hospital building in Banfora was converted to a clinical trial facility, where all CNRFP clinical trial activities are conducted. Elsewhere, we are analysing the operation of these research centres and the implications of such long-term engagements. The focus of this paper is health providers’ and managers’ perceptions of the impact of on-going malaria vaccine trials (MVTs) on routine health service delivery in facilities where MVTs were being implemented.

At the time of the study, the KHRC-Ghana and KWTRP-Kenya were conducting RTS,S phase III malaria vaccine trials while the CNRFP in Burkina Faso was undertaking a GMZ2 phase II malaria vaccine trial. These trials involved assessing the safety and efficacy of vaccines against malaria among infants and children [42, 43]. They were conducted in rural government health facilities including community clinics, dispensaries, health centres and referral hospitals and involved existing Ministry of Health (MoH) staff as well as specifically recruited trial staff (see Table 2). In Ghana and Burkina Faso the MVTs were conducted in facilities that had been involved in previous research centre activities. By contrast, in Kenya all of the MVT activities (apart from the provision of clinical care to referral patients) were conducted in health facilities with no previous involvement with research centre activities (Table 2). In all three countries a range of stakeholders were consulted by MVT principal investigators, to assist in selecting the health facilities to be involved in the trial, and to discuss the types of resources required for MVT implementation in the selected facilities. Discussions were also held to identify how MoH staff working in the selected facilities might become involved in the MVTs and how such staff would be supported.

**Table 2:** Description of the malaria vaccine trials.

Trial details	Ghana: RTS,S phase III malaria vaccine trial	Kenya: RTS,S phase III malaria vaccine trial	Burkina Faso: GMZ2 phase II b malaria vaccine trial
Trial duration	March 2009 – January 2014	May 2009 – January 2014	April 2011 – July 2013
Children recruited (age/number)	1,333 healthy children: 5–17 months (n = 1002), 6–12 weeks (n = 331)	904 healthy children: 5–17 months (n = 600), 6–12 weeks (n = 304)	580 healthy children:12–60 months (n = 580)
Trial location	Kintampo North and Kintampo South Districts	Ganze District, Kilifi County	Banfora District
Government facilities involved; distance from clinical trial facility (km/m)*	3 health centres: 19 km, 47 km and 51 km from KHRC	2 dispensaries; 27 km and 38 km from KWTRP	- 2 community clinics (CSPS)^a^; 5 km and 7 km from CRUB^b^
	Kintampo North Municipal hospital: 50 m from KHRC	1 health centre; 30 km from KWTRP	
		1 county hospital; same location with KWTRP	
Role of **primary care facilities** in the MVT	**3 health centres:** recruitment sites; patient monitoring; provision of patient care during and outside working hours; *MVT vaccination centre*	**1 health centre and 2 dispensaries:** recruitment sites; patient monitoring; provision of patient care during and outside working hours; *MVT vaccination centre*	**2 community clinics:** recruitment sites; patient monitoring; provision of patient care during and outside working hours; *station for picking and dropping participants for scheduled visits at the CRUB*
Role of **tertiary/ referral facilities** in the MVT	**Kintampo North Municipal hospital:** study participants referral and in-patient care; provide oversight and support for existing MoH staff in MVT; *MVT vaccination centre*	**Kilifi County hospital:** study participants referral and in-patient care; provide oversight and support for existing MoH staff in MVT	**CRUB:** study participants referral and in-patient care; provide oversight and support for existing MoH staff in MVT; *MVT vaccination centre*
Facilities with **prior research/CT** involvement	Kintampo North Municipal hospital, conducted other inpatient CTs	Kilifi County Hospital, conducted other inpatient CTs	CRUB, conducted other malaria vaccine and drug CTs
	1 health centre, site within HDSS		2 community clinics, sites within HDSS
Facilities with **no prior research/CT** involvement	2 health centres, sites within HDSS	1 health centre and 2 dispensaries, sites outside HDSS	

*km/m: kilometre/metre;

^a^ CSPS: Centre de Santé et de Promotion Sociale;

^b^ CRUB: clinical research unit of Banfora.

The principal investigators (PIs) of the malaria vaccine trials in Ghana and Burkina Faso were also the PIs for this current study of the effect of the trial activities on provider perceptions and practices, but the study activities were developed and implemented by the social science research teams at each site. In Kenya, the PIs for this study were not members of the malaria vaccine trial, but were from the same institution, the KWTRP.

## Methods

An initial partners’ meeting was held in Kintampo, Ghana in March 2012 to develop a common study methodology across all three sites to allow for inter-site descriptive comparisons. To describe the malaria vaccine trial inputs and explore their impact on the perceptions and practices of these health managers and health care providers we employed both quantitative (health facility and human resources audits) and qualitative (in-depth interviews and focus group discussion) methods. Data were collected between May 2012 and June 2013 by teams of trained research officers and fieldworkers in each country using the agreed common data collection techniques. Sampling procedures were purposive to ensure inclusion of people of different cadre, level of involvement in trial activities and demographic characteristics. The data sources, sampled facilities and characteristics of interviewees in the three countries are described in Table 3.

**Table 3:** Summary of data collected.

Method	Respondent type	Ghana		Kenya		Burkina Faso		Total
		Non-MVT	MVT	Non-MVT	MVT	Non-MVT	MVT	
Facility audits^a^	CT facilities	0	4	0	3	0	3	**10**
Human resource audits	MoH staff	5	10	3	6	0	0	**24**
	Research centre staff	23	24	0	5	25	43	**120**
Investigators interviews	Senior CT investigators	0	1	0	1	0	10	**12**
Health providers interviews	MoH staff	9	2	3	6	10	19	**49**
	MVT clinicians	2	14	0	5	3	6	**30**
	MVT fieldworkers	0	0	0	0	0	8	**8**
Health managers interviews	District health managers	4	0	0	0	2	0	**6**
	Regional health managers	2	0	0	0	2	0	**4**
	National health managers	0	0	0	0	4	0	**4**
Focus group discussion	MVT fieldworkers	3	3	0	3	0	0	**9**

MVT: Malaria vaccine trial

^a^ MVT facilities audited include a referral hospital (Ghana = 1), health centres (Ghana = 3; Kenya = 1), dispensaries (Kenya = 2), community clinics (Burkina Faso = 2) and a clinical trial facility (Burkina Faso = 1).

### 2.1. Health facility and human resource audits

In each country, all of the facilities (n = 10) involved in the MVT were included in the health facility audits. Each facility was visited by the research team and a structured checklist was used to collect data on the presence, functional status (on day of survey) and funding source of laboratory and clinical care equipment, as well as facility infrastructure (such as buildings and vehicles). The human resource audit (n = 144) covered all of the facilities involved in the MVTs and involved a purposefully selected sample of staff to represent the range and type/cadre of all staff present in each facility, and research centre staff involved in clinical trials. Data were collected on their primary and secondary responsibilities and whether these were linked to the malaria vaccine trial, and the type and nature of training received.

### 2.2. In-depth interviews and focus group discussions

In-depth interviews (IDIs) were undertaken with a range of health care providers and managers to investigate their experiences and perceptions of the impact of clinical trial activities on the quality of health care provided at the health facilities. Issues around MVT-MoH linkages and perceptions of post MVT impact were also explored. IDIs were held with senior investigators (n = 12), front-line health workers (n = 87) and health mangers (n = 14). All senior investigators with at least one year’s involvement in planning and implementation of the malaria vaccine trials were approached for interviews, and frontline health workers were purposefully selected based on their mechanism of employment (MoH staff involved in the trial, MoH staff not involved in the trial, and MVT employed staff) and their cadre (clinicians, public health officers, fieldworkers and support staff). We also approached health managers and policy makers at district, regional and national level with relevant health programme planning and implementation roles, such as Regional hospital mangers, District Health Management Team (DHMT) members and Maternal and Infant Health programme directors—see Table 3. In addition, in Kenya and Ghana focus group discussions (n = 9) were conducted with the fieldworkers (employed by the trial to conduct follow-up home visits and liaise between the health facility and community) in order to gather their views on how the trial inputs affected the functioning of the health facilities.

### 2.3. Data management and analysis

Each site was responsible for their own data collection and management. For qualitative data, verbatim transcriptions and back translations were undertaken by the study team in each country. Across sites the data were managed in NVivo 8 and analysed using a framework approach [44, 45]. This process involved in-depth reading of transcripts to identify emerging themes across the datasets, developing a coding framework to code data, generating charts to summarise the data by categories, and data interpretation, to identify differences/similarities and provide explanations of the analysed data. The quantitative audit data were collated by the study teams in each country, and entered into Microsoft Excel to generate descriptive summaries.

To facilitate cross-site synthesis and comparison, empty analysis tables were developed on the basis of an in-depth discussion of preliminary results from each site (one week workshop in Ghana). Each site then pulled out all data—qualitative and quantitative—to populate these tables. In a second workshop in Kenya, the data for each site were then collated and discussed. We employed the World Health Organisation (WHO) health systems building blocks to aid in the development of our framework for analysis and tables, and in the interpretation of data [46].

### 2.4. Ethical consideration

Ethical approvals were obtained from national ethics committees of the respective countries prior to the commencement of study activities (Ghana Health Service and Kintampo Health Research Center in Ghana; KEMRI National Ethical Review Committee in Kenya; Comité d’Ethique sur la Recherche en Santé (CERS) and Comité de Bioéthique Institutionnel du CNRFP (CIB/CNRFP) in Burkina Faso. Separate local ethics clearances were also obtained at each site. All data were anonymised, with access limited only to researchers. Written informed consent was obtained from all interviewees. Prior to any data collection, the malaria vaccine trial staff were briefed about our study, and permission was sought to conduct the work from health facility staff and opinion leaders. We gave feedback to malaria vaccine trial staff on emerging issues of importance and shared preliminary results with the MVT teams.

## Results

### 3.1. Inputs to MVT facilities and perceived impact on routine service delivery

In all three countries, establishing the MVT sites (within local health facilities involved upgrading and renovating the physical infrastructure, providing medical supplies and equipment, increasing human resources, providing additional funds and training and supporting MVT participants’ medical costs.

#### i. Physical inputs to facilities

**Physical infrastructure.** In Ghana, the Kintampo North Municipal hospital and one of the three health centres had previously been involved in other KHRC research activities. During the RTS,S phase III trial set-up, the children’s ward in the referral hospital and child welfare clinics in the three health centres were repainted and the patient waiting bay area expanded. In Kenya, the Kilifi County Hospital receives on-going support from the KWTRP but the 3 rural facilities selected for the RTS,S phase III had minimal prior engagement with the KWTRP research activities. At the time of MVT set-up, upgrades to these rural facilities included the construction of additional rooms, expansion of patient waiting bays, new roofing and repainting. In Burkina Faso, the Clinical Research Unit of Banfora (CRUB) has continued to receive support from pooled funds from the programme to centrally run its clinical trial activities. During MVT set-up, the CRUB was entirely refurbished and fully equipped by the CNRFP, although no upgrades were undertaken in the community clinics, which served as recruitment sites (Table 4).

**Table 4:** Physical inputs brought by MVTs.

Inputs	Ghana	Kenya	Burkina Faso
**Physical infrastructure**	1 children’s ward and 3 child welfare clinics renovated	2 rooms and 1 extension constructed	6 rooms (office) constructed
	4 off-road vehicles	3 off-road vehicles	9 off-road vehicles
	3 plastic water tanks	1 plastic water tank	1 plastic water tank
	2 generators		1 generator
	35 Beds		6 Beds
**Perceived changes due to MVT inputs**	MVT off-road vehicles facilitated emergency referrals, ferrying of medical supplies and patient access to facilities	MVT off-road vehicles facilitate emergency referrals, ferrying of medical supplies and patient access to facilities	MVT off-road vehicles facilitate emergency referrals, ferrying of medical supplies and patient access to facilities
	Installed generators provide additional power source to the referral hospital	Additional MVT constructed rooms created more space and facilitated provision of other routine services e.g. vaccination and maternal deliveries	Installed generators provide additional power source to the trial facility
**Perceived concerns/ challenges**		In smaller facilities, limited space and capacity for both MVT activities and routine services	Having MVT activities separated from routine care, limits access to enhanced services by community members not in MVTs

Across all three countries the renovations undertaken to support the MVT were appreciated by frontline health staff. Some respondents said that the additional rooms provided extra working space that could be used during and after MVT activity hours, and that upgrades of existing structures (such as the children’s wards and patient waiting bays) created a conducive environment for providers and health facility users. In Kenya for instance, one health care provider said that during MVT set-up, the facility management requested the MVT team to have one of the newly constructed rooms converted to a facility delivery room, and this was perceived to contribute to improved maternal deliveries and staff performance.

*…when the trial was starting I had a big problem with finding a place to do deliveries for pregnant mothers*, *and as government employees we are gauged on our performance annually on certain services…when the trial came we requested they construct for us a room…and it has helped because the community benefits and I am able to report to my employer… (* ***IDI11/MoH with top-up/Kenya*** *)*

However, some of the respondents reported concerns linked to the physical location of MVT activities and capacity within facilities. For instance, in smaller facilities there were concerns of insufficient space for both trial and routine service delivery, potentially leading to over-crowding, as expressed by this respondent from Kenya:

*…in the same location we [MoH staff] do pregnancy screening*, *they [MVT team] sometimes use the room for blinding [investigational product concealment] and administer vaccines*. *When a client needs VCT [voluntary counselling and testing] we [MoH staff] use the same room…and yet the same room is our [facility] delivery site… (* ***IDI16/MoH with top-up/Kenya*** *)*

Another challenge articulated by some participants was that while there were efforts to ensure MVT activities were laid out to blend with existing structures not all were successful. For instance, in Burkina Faso there was a concern that the separate construction for MVT activities limited the access to these enhanced health services for community members not enrolled in the trial.

**Transport infrastructure.** Across all three countries MVTs purchased additional off-road vehicles (ranging between 2 and 9 cars) to facilitate MVT activities at the peripheral MVT health facilities. Although availability of MVT transport was mainly to facilitate the implementation of MVT activities, the majority of frontline providers across the three countries felt the presence and access to MVT vehicles played a big role in supporting routine services. For instance, transportation of essential drugs and other medical supplies, improved patient access to facilities (allowing for early diagnosis and treatment of illnesses) and assisted with patients’ referral and community outreach. These inputs were perceived to benefit MVT participants and non-participants alike.

**Medical care support.** Across all three countries, the MVTs channelled substantial funding into the purchase of medical equipment such as laboratory equipment, oxygen cylinders and examination couches (Table 5). The MVTs ensured there were regular supplies and access to essential drugs (such as paracetamol, antibiotics, antimalarials and cough syrup) at MVT sites and clinical equipment (such as thermometers, anthropometric scales, and stethoscopes) where needed for routine patient clinical care (Table 5). Audit data indicate that in Ghana and Burkina Faso, hospitals and clinical trial units received more clinical care equipment than peripheral health facilities; by contrast in Kenya, there was more support to dispensaries and health centres. This difference is likely to have been due to the different roles of these facilities in MVT related activities and the additional inputs to facilities to help meet MVT site standards.

**Table 5:** Medical care support to MVT facilities.

Inputs	Ghana	Kenya	Burkina Faso
**Medical equipment (selected items)**	3 Oxygen cylinders & gas	3 Oxygen cylinders & gas	2 Oxygen cylinders & gas
	5 Stethoscopes	6 Stethoscopes	5 Stethoscopes
	50 Thermometers	32 Thermometers	18 Thermometers
	2 Length boards (<5 years)	3 Length boards (<5 years)	5 Length boards (<5 years)
	5 Children weighing scales	6 Children weighing scales	5 children weighing scales
	3 Examination coaches	3 Examination coaches	2 Examination coaches
	2 Pump and suction machine	2 Pump and suction machine	1 Pump and suction machine
	8 Manual suction apparatus	2 Manual suction apparatus	1 Oropharyngeal tube
	2 Ambu bags	1 Ambu bag	1 Laryngoscope
	2 Cardiac monitors	2 Oropharyngeal tubes	1 Cardiac monitor
	1 Digital X-ray machine and printer	1 Laryngoscope	
**Perceived changes of procedures/ routine services due to MVT**	Provision of diagnostic kits supported with training helped improve malaria case management	Provision of diagnostic kits supported with training helped improve malaria case management	Provision of diagnostic kits supported with training helped improve malaria case management
	Access to equipment for improving emergency care in rural facilities	Access to equipment for improving emergency care in rural facilities	Access to equipment for improving emergency care in rural facilities
	Improved radiology services in hospitals for all users	Improved radiology services in hospitals for all users	
	Standard clinical practise to screen all febrile children for malaria and bacteraemia on admission, in all MVT facilities		

Most MoH health providers appreciated the improved access to essential drugs provided by the MVTs. Some MoH staff said the availability of malaria diagnostic kits and training in their use help them adhere to treatment guidelines rather than treating malaria presumptively, while others suggested there had been a general reduction in malaria morbidity and mortality due to MVT activities.

*…were it not for the study child [participant]*, *you would have treated them clinically [presumptively]…but because we have been given kits [malaria RDTs]*, *you test them and at least you feel that you have done something good*. ***(IDI20/MoH with top-up/Kenya)***

In addition, in the past most facilities had challenges handling emergency care, hence the availability of oxygen cylinders and gas donated by MVTs to peripheral and tertiary health facilities, was reported to enable clinicians help resuscitate sick patients. Furthermore, in Ghana the availability of digital x-rays was perceived as a major boost to the hospital and due to the presence of MVT activities, it became standard practice to screen all febrile children for malaria and bacteraemia on admission in all MVT facilities.

#### ii. Human resource inputs to facilities in each country

**Staffing.** In all three countries additional full time staff were hired during the MVT period to assist with patient clinical care at the MVT peripheral health facilities (Table 6). These included study clinicians (doctors, nurses and clinical officers) who were hired to provide clinical care and administer MVT vaccines to study participants; and fieldworkers, who were employed from local communities where MVTs were conducted to assist in conducting follow-up home visits. In MVT facilities, existing MoH clinical staff were also employed on a locum basis, or provided salary top-ups to assist with the provision of clinical care, especially to study participants during and out-of facility working hours.

**Table 6:** Human resource input by MVTs.

Inputs/ incentives	Ghana	Kenya	Burkina Faso
**Full time MVT staff (primary care facilities)**	114 project staff (13 doctors, 60 fieldworkers)	59 project staff (2 doctors, 5 clinical officers, 4 nurses, 29 fieldworkers)	68 project staff (10 doctors, 12 nurses, 16 fieldworkers)
**MoH staff paid MVT salary/top-up (primary care facilities)**	23 MoH staff (nurses and community health officers)	12 MoH staff (1 clinical officer, 11 nurses)	20 MoH staff (16 nurses, 4 Lab assistants)
**MoH staff paid MVT salary/top-up (referral hospitals)**	13 MoH staff (paediatricians, doctors and other health professionals)		2 MoH staff (Paediatricians/doctors)
**MoH staff *not* paid MVT salary/top-up (primary care facilities)**	80 MoH non-clinical staff	26 MoH staff: Public Health Officers, Pharmacy aide(s), support staff	6 MoH staff: pharmacy aide(s), guards
**MoH training and who considered (primary care facilities)**	GCP, GLP and vaccine cold-chain management; MoH clinical staff	GCP, GLP, communication and research ethics; MoH clinical staff	GCP and GLP; MoH clinical and non-clinical staff
**Perceived changes of procedures or routine services due to MVT (primary care facilities)**	Presence of MVT activities led to extended facility operational hours	Presence of MVT activities led to extended facility operational hours	Presence of MVT activities led to extended facility operational hours
	Constant availability of qualified medical staff improves disease surveillance and facilitate learning (for MoH staff)	Constant availability of qualified medical staff improves disease surveillance and facilitate learning (for MoH staff)	Constant availability of qualified medical staff improves disease surveillance and facilitate learning (for MoH staff)
	MoH clinicians trained on cold-chain management able to support MVT vaccination	MVT vaccines administered in the same location with routine childhood vaccines, strengthening integration and learning. MVT clinicians assist with routine childhood vaccination	
**Perceived concerns/ challenges**	Additional workload due to MVT activities—filling MVT forms, working extra hours, providing back-up roles without pay (MoH non-trial staff)	Additional workload due to MVT activities—filling MVT forms, working extra hours, providing back-up roles without pay (MoH non-trial staff)	Additional workload due to MVT activities—filling MVT forms, working extra hours, providing back-up roles without pay (MoH non-trial staff)
	Some MoH non-trial staff feel excluded in consideration of MVT training and allowances	Some MoH non-trial staff feel excluded in consideration of training and allowances	MoH staff lack supportive supervision once trained, need additional training to handle MVT purchased medical equipment, and high-staff turnover requires constant further training for newly posted MoH staff

* GCP; Good clinical practice; GLP: Good Laboratory Practice—including training in microscopy use; MoH: Ministry of Health.

Across all three countries, the majority of respondents appreciated the additional health workers employed by MVTs, as they were perceived to contribute to a reduction in workload for MoH health workers. For instance, MVT clinicians supported with all paediatric patient consultation during busier clinic days in MVT primary care facilities or while the nurse in-charge/MoH clinicians were absent from the facility (while attending official meetings—all three countries, and during the nationwide health workers strike—Kenya only). In Kenya and Ghana, MVT clinicians regularly supported MoH clinicians with the administration of routine childhood vaccines alongside MVT vaccines.

*…we [MoH staff] are over-stretched and we don’t have a lot of staff; so when they [MVT staff] go [to health facilities]*, *they also assist with some of the routine aspects of the OPD [out-patient department] cases and other cases*. *We are able to take lessons and pass it on to the other [MoH] nurses…* ***(IDI/MoH without top-up/Ghana)***

The facility staff and some trial frontline staff also reported that MVT staff at MVT peripheral facilities provided supportive supervision, acted as role models and could easily be approached to seek clinical advice/consultation. Most of these respondents felt the good working relationship that existed between trial and MoH staff facilitated a good learning environment.

*With the presence of CNRFP workers [nurses] in the facility*, *the behaviour of other health workers has changed*. *They are more attentive towards patients and are more careful in filling in [information in clinic] books*, *even when they are not those CRFs [case report forms] of CNRFP*. *(* ***IDI44/MoH without top-up/Burkina Faso*** *)*

By contrast, across all three countries, some staff (among all cadres and types of employment) felt MVT activities had led to an increase in their workload, through adding new activities such as filling in MVT forms, having to work for extra hours, and performing extra chores without pay. The latter concerns were raised particularly by trial fieldworkers and MoH non-trial staff.

**Training.** In all three countries, the MVTs provided training in good clinical practice (GCP), good laboratory practice (GLP) and health research ethics (HRE). However, the training provided varied across the countries by cadre of MoH staff and according to level of involvement in trial related activities. For instance, in Ghana and Kenya, MoH clinical staff involved in trials were provided with training but non-clinical staff were excluded. By contrast, in Burkina Faso, all staff at the community clinics involved in the trial received training. In most instances, the majority of the MoH frontline providers had minimal prior research experience and the training provided by the MVTs were organised during site initiation and when new MoH staff were posted to MVT facilities. In general, access to training was perceived to be an individual benefit and a benefit for the patients through improvements in the quality of care provided.

*All the staff involved with patient care in our health facility were trained on microscopy use…and how to perform RDTs [rapid diagnostic tests]*. *In district meetings*, *we [MoH staff in MVT facilities] are considered privileged…* ***(IDI40/MoH with top-up/Burkina Faso)***

However, there were mixed perceptions regarding the nature, practicality and relevance of the MVT related training. For instance, participants in Burkina Faso expressed concern that staff were trained on GCP but lack supportive supervision and in Kenya one of the respondents found the training ‘complex’ and would need to reconsider attending a refresher course. In Burkina Faso concerns were raised about the amount of training needed to handle new MVT purchased medical equipment (such as haematology analysers), particularly since high staff turn-over invariably meant further training for newly posted MoH staff.

#### iii. Health financing and perceptions of input

**MoH staff incentives.** In all three countries, the MVTs provided salary top-up or allowances to existing MoH staff (including paediatricians, nurses, doctors) at peripheral and referral health facilities to support various aspects of MVT activities (see Table 6). For instance health managers provided oversight and support supervision to MoH staff in MVT facilities, while MoH clinicians provided care to study patients during and out-of work hours. However, the calculation of payment varied by country. In Ghana, it was based on number of hours worked and in Kenya, it was calculated as a fixed proportion of the monthly salary. In Burkina Faso, it was based on involvement in specific trial activities. There were also country variations in who was eligible for a salary top. For instance, in Ghana and Kenya only MoH staff providing clinical care were included while in Burkina Faso, all MoH staff, irrespective of their cadre/specialization were included.

*… the project [MVT] pays monthly to the facility the amount corresponding to the total number of filled files*. *Then the in-charge redistributes this amount to all facility staff so that each staff gains something*, *even the guard and the essential drugs deposit staff*. *This [approach] seems more equitable to us [facility staff]*. *(* ***IDI31/MoH with top-up/Burkina Faso*** *)*

The mechanism of rewarding staff generated mixed reactions. In all three countries, most MoH clinical staff receiving a salary top-up reported that their standards of living had improved (particularly compared to their MoH colleagues not involved in MVTs) and they felt motivated to perform better. These sentiments were echoed in interviews with trial staff.

*Regularly giving them “something” [top-up] motivates MoH staff in the peripheral health facilities participating in CTs*. *Not only do they work well*, *but they also work regularly…the output is quite different… (* ***IDI01/trial staff/Burkina Faso*** *)*

However, MoH frontline staff in all three countries also reported challenges with the MVT reward mechanisms. Some of the MoH clinical staff receiving a top-up felt their pay compared poorly to trial clinicians even though they performed similar roles. Many MoH staff not receiving a top-up were unhappy that they were excluded from this benefit despite providing back-up to their colleagues where needed.

**Facility operational cost.** The MVTs covered some of the operational costs incurred by the MVT peripheral health facilities including a monthly fuel and communication allowance in Ghana and Kenya, and an internet connection to the referral hospital in Ghana and the CRUB in Burkina Faso. These inputs improved the connectivity of the peripheral facilities, enhancing their ability to manage their drugs supplies and obtain support in clinical decision making. In Kenya, the dispensaries were able to charge the MVT for the user-fee payments that they would have otherwise received from the MVT participants when they visited the facility. Given the relatively high use of facilities by trial participants (encouraged by the MVT staff) these funds contributed to increased facility funding, allowing for the payment of support staff salaries and the purchase of supplies like gloves.

*…so in one of the dispensaries…we [MVT and facility staff] had to agree that any participant seen will be recorded and pay their user fee on a monthly basis…so it’s a small income for the dispensary*…*and they use the money to pay subordinate staff and sustain services within that dispensary*. ***(IDI24/trial staff/Kenya)***

### 3.2 What happens when MVTs are concluded

In interviews with health workers and managers, we explored their perceptions of the potential impact of the ending of the MVTs and the withdrawal of staff and other inputs on staff and service delivery in facilities where MVTs were implemented. We discuss their views and suggestions for MVT exit.

#### i. Equipment and infrastructure

The participants were ambiguous about the potential post-trial benefits and challenges associated with the equipment and infrastructure brought to the facilities by the MVTs. Many were unsure of what would be left behind and, if equipment was left behind, who would be responsible for maintenance. In all three countries concerns were raised over MoH capacity (financial and technical) to handle and maintain equipment after trial closure.

*…these days facilities don’t generate income and it would be very difficult for them to take good care…or provide maintenance to their structures…*.*any money that comes to the system is programmed*…*so getting funds to maintain the clinical trial infrastructure would be difficult*. ***(IDI/MoH without top-up/Ghana)***

In addition, some MoH frontline staff suggested that before the closure of the trial consideration should be given to providing additional support such as facility upgrades, or donation of funds to a facility kitty to assist with facility operations post-trial.

#### ii. Staffing and employment

In all three countries, fieldworkers and MoH clinicians reported concerns about loss of employment and earnings, with negative implications for staff morale and facility workload.

*…there will be a very big gap*. *The cadre of professionals who were working here…like medical officers at dispensary level is something that rarely happens in our country…we get a lot of assistance from them [trial]… (* ***IDI16/MoH with top-up/Kenya*** *)*

#### iii. Facility service delivery

It was universally perceived that withdrawal of MVT staff and services (transport and drug supply) would lead to a significant deterioration in facility services including longer queues/waiting time, increased MoH staff workload, and a return to drug-shortages and problems with facility access.

*When the study leaves our hands will be chopped*! *Because when we ran out of supplies*, *they [MVT] bring for us…*.*if they leave we will suffer…* ***(IDI20/MoH with top-up/Kenya)***

Other dilemmas expressed especially by fieldworkers and MoH staff were how to handle community expectations. In particular, they felt participants and their families would face problems meeting their health care costs, and that this could impact on their treatment seeking patterns.

*… Mothers will go back to their initial habits of delaying to seek care in facilities when their child is sick*, *because they lack money to buy drugs…when the trial ends and there is no free care*, *mothers will opt to stay at home when their children are sick*. *(* ***IDI28/trial staff/Burkina Faso*** *)*

## Discussion

The potential benefits and consequences of clinical trials on communities and facilities in which they are implemented has led to calls for more empirical work to understand how CTs impact health service delivery in Africa. In this paper, we draw on quantitative and qualitative data from studies of MVTs in Ghana, Kenya and Burkina Faso to explore health providers’ perceptions of the impact of malaria vaccine trials on routine health service delivery, and the quality of care provided.

There was considerable heterogeneity in the way in which trial centres were established and in the implementation of the malaria vaccine trials across the study sites and trials. However, all three trials provided considerable inputs into the public health facilities in which they were based. Our findings, in common with those of other studies [9, 10, 13, 18, 20], suggest that CTs have the potential to benefit facilities through infrastructure upgrades, the provision of medical care support and additional qualified personnel, and by supporting some facility operational costs. For instance, the availability of transport provided by the trials supported facility referral services, the increased interconnectivity allowed for support to decision making and increased opening hours with extra staff allowed for improved patient access to health care. The constant availability of essential drugs also helped to address the drug shortage problems faced in many sub-Saharan African settings.

Across all the three countries, the MVTs provided salary top-up and training to existing frontline staff to assist with MVT activities and provide routine care. The MoH staff perceived that the additional wages helped them improve their standard of living and motivation. However, our findings also suggest that all of the different reward mechanisms employed across the countries (only paying MoH clinical staff in Ghana and Kenya versus paying all facility staff irrespective of their cadre in Burkina Faso) generated dissatisfaction and relationship challenges among some frontline staff, and had the potential to demotivate those staff not involved in the process. Similar dilemmas were reported in a Cameroon trial on the use of mobile phone reminders among HIV patients receiving treatment from a referral hospital [13]. In a context where facility staff wages are often low, several authors have suggested that to avoid perverse outcomes, CTs need to ensure that the reward mechanisms are fair and considered alongside other non-financial incentives such as training and improved working environment. These authors have also pointed out that any rewards have to be balanced against the risk of escalating trial budget costs and the potential to undermine more intrinsic forms of health worker motivation such as altruism and social recognition [9, 12, 13, 24]. The data from this study suggest that even if negative effects of these rewards are not experienced at the time of the trial, their withdrawal once the study has concluded could have harmful effects on staff morale and ultimately patient care.

Uncertainty over what would happen once the MVTs were concluded was an emerging concern among participants across all three countries. The large majority of participants were of the opinion that the ending of the trial would have a negative impact on staff through loss of wages, with facility services deteriorating due to a recurrence of drug shortages, lack of transport for referrals and increased workload due to the removal of additional staff. There were also concerns about whether or not MVT equipment and infrastructure would be left behind and, if it were, who would be responsible for its maintenance. Such fears have been found in other studies of the impact of CTs [8, 11, 47] suggesting a lack of capacity and confidence among MoH implementers to maintain some of the positive changes experienced during trial period. There are particular concerns if there is a potential for remaining staff and facilities to be left ‘worse off’ than before the trial began, as a result of raised expectations being dashed, or new non-essential costs being introduced that cannot be maintained.

In addition to the infrastructural inputs, CTs invest in training and capacity building of frontline staff, to ensure that standards of care provided meet local and international standards [1, 6, 7, 11]. In this study we found that, on the whole, the training provided by the MVTs was perceived to be beneficial. However, across the countries different strategies were adopted for the selection of staff for training and staff excluded from training expressed concerns about their omission. These feelings of marginalization were particularly acute among non-clinical staff and may impact on performance. A recent study in Kenya found that a lack of fairness in attending seminars or other training opportunities, and limited opportunities for career advancement, affected health workers performance and motivation [48]. In our study, among the MoH staff who had received GCP training there was also dissatisfaction with the lack of subsequent supportive supervision, although training was provided for any new staff who joined the clinic during the lifetime of the MVT. This happened fairly frequently because, as elsewhere in sub-Saharan Africa [24, 49], staff turnover in rural health facilities in all three countries was high. Our data suggest that CTs should carefully consider the nature of the training that they provide to trial and facility staff. Training that is relevant to service provision for all users of trial facilities—including in technical care provision but also, for example, in governance and oversight of facilities—has the potential to have a positive impact on a wide range of users and the staff themselves in their current and future careers.

The inputs and strategies employed by the three MVTs involved in this study to ensure the provision of quality health care to trial participants were, perhaps unsurprisingly, very similar to the health system strengthening interventions implemented in many countries in sub-Saharan Africa in an attempt to improve the quality of patient care. Data from evaluations of such interventions suggest that continuous education and training are critical in motivating frontline staff and improving performance [3, 12, 24, 49] but that any improvements disappear quickly if no follow-up support is provided [50–55]. Furthermore, recent research suggests that changing health worker practices requires the development and maintenance of a supportive community of practice [56, 57]. Our data suggest that the presence of MVT clinicians in the health facilities for the duration of the trial and the enhanced communication facilitated by the trial inputs and activities helped to provide a community of practice that enabled non-trial clinicians to improve the quality of care they provided. However, our interview data suggest that the non-trial clinicians had little confidence in their ability to maintain these practices once the trial concluded and the ‘trial clinician community’ was disbanded.

CTs may, in the short term and for the duration of the trial, be able to provide the elements and conditions (such as training, support supervision, enhanced community of practice, and supplies of drugs) under which frontline health workers are able to improve their practices. However, as the data from this and other studies suggest, maintaining new practices requires system related changes that lie outside the realm of individual health workers or health facilities [51, 56–59], and many complex interventions have been developed and implemented over recent years in an attempt to facilitate such change, with varying levels of success [51, 52, 55, 60, 61]. We would argue that while CTs mimic the inputs of health system strengthening interventions, they are not designed as such and cannot take on this role. On the other hand, where they do operate alongside the frontline provision of care, trialists need to be aware of their potential impact on health worker motivation and behaviour and should take note of the lessons provided by the health systems strengthening literature in the design of strategies for CT implementation.

### Recommendations for future clinical trials

While CTs are clearly not designed as health system strengthening interventions we suggest that there are steps that trials can take to help ensure that they leave behind positive rather than negative impacts. We suggest the need for CTs to ensure they have a clearly documented memorandum of understanding (MoU) with MoH managers and facility staff at the onset to identify the level of CT support required, and develop a strategy for staggered exit and handover of responsibilities and equipment to MoH or other key local actors when CTs end. Such a process could help identify each stakeholder’s responsibility including maintenance issues and should include a process of negotiation to handle any unmet expectations. In the early post trial period, CTs could consider maintaining some staff to help monitor and provide support in addressing post-trial issues. Careful documentation and evaluation of different exit strategies would be valuable to the planning of future trials, while recognising the limits of the health delivery responsibilities that trials can and should take on during and after trials. The latter is particularly important given both the need for locally appropriate, ethically conducted, trials in the region, and the potential for escalating costs and bureaucratic requirements to undermine the ability of such trials to be conducted.

### Limitations

The focus of this study was malaria vaccine trials based in peripheral health facilities and district hospitals. While some of the inputs recorded are likely to be specific for malaria prevention or treatment trials (e.g. antimalarial drugs and malaria rapid diagnostic tests), our long experience of clinical trials based in similar settings in sub-Saharan Africa suggests that many of the key inputs, such as salary top-ups for staff, staff training, enhanced communication, and physical infrastructure improvements, are common across a range of clinical trial types. Furthermore, our findings echo those from other studies on the impact of individual clinical trials and, in this study, the use of similar tools across heterogeneous contexts (country, research centres and type of facilities) and diverse respondents (trial staff, MoH frontline providers and health managers) has enabled us to paint a broad description of health provider perceptions of the effects on service provision of hosting a clinical trial in low resource settings. A potential constraint in data collection (particularly the in-depth interviews) was the association between the study research teams and the clinical trial teams in each country. The study research teams were from the same institution as the trial implementing teams and, while the research team were independent of the trial team, it is possible that knowledge of this relationship influenced interview responses.

## Conclusion

Clinical trials inputs have the potential to improve health service delivery and benefit the facilities, staff and communities involved in clinical trials. However, there are limits to the wider health system benefits that can be expected and achieved through trials and unexpected and unintended negative consequences can occur both during the lifetime of the trial and after the trial has been completed. While clinical trials should not be expected to fill the role of health service strengthening interventions, our findings suggest that to strengthen ethical practice, consideration of some of the potential benefits and harms to the wider health system need to be considered from the outset and discussed with key local stakeholders and funders. Such discussions are likely to need to be revisited and revised throughout the trial, as well as immediately post completion, as part of wider trial planning and community engagement strategies, and in response to emerging issues.
